# An Association between Single Nucleotide Polymorphisms of Lys751Gln *ERCC2* Gene and Ovarian Cancer in Polish Women

**DOI:** 10.1155/2015/109593

**Published:** 2015-10-07

**Authors:** Magdalena M. Michalska, Dariusz Samulak, Hanna Romanowicz, Maciej Sobkowski, Beata Smolarz

**Affiliations:** ^1^Department of Obstetrics and Gynaecology, Regional Hospital in Kalisz, Poznańska 79, 62-800 Kalisz, Poland; ^2^Cathedral of Mother's and Child's Health, Poznan University of Medical Sciences, Polna 33, 60-535 Poznań, Poland; ^3^Laboratory of Cancer Genetics, Department of Pathology, Institute of Polish Mother's Memorial Hospital, Rzgowska 281/289, 93-338 Łódź, Poland; ^4^Department of Obstetrics and Gynaecology, University Hospital, Polna 33, 60-535 Poznań, Poland

## Abstract

*Aim.* The aim of this study was to evaluate the role of the Lys751Gln (rs13181) *ERCC2* gene polymorphism in clinical parameters and the risk for development of ovarian cancer. *Material and Methods.* The study consisted of 430 patients with ovarian cancer (mean age: 53.2 ± 10.11) and 430 healthy subjects (mean age: 50.31 ± 18.21). Analysis of the gene polymorphisms was performed using the PCR-based restriction fragment length polymorphism (PCR-RFLP). The odds ratios (ORs) and 95% confidence intervals (CIs) for each genotype and allele were calculated. *Results.* The results obtained indicate that the genotype Gln/Gln is associated with an increased risk of ovarian cancer (OR 5.01; 95% CI 3.37–7.43; *p* < 0.0001). Association of Lys751Gln polymorphism with histological grading showed increased *ERCC2* Gln/Gln (OR = 6.96; 95% CI 3.41–14.21; *p* < 0.0001) genotype in grading 1 as well as Gln allele overrepresentation (OR = 4.98; 95% CI 3.37–7.40; *p* < 0.0001) in G1 ovarian patients. Finally, with clinical FIGO staging under evaluation, an increase in *ERCC2* Gln/Gln homozygote frequencies in staging I and Gln allele frequencies in SI were observed. *Conclusion.* On the basis of these results, we conclude that *ERCC2* gene polymorphism Lys751Gln may be associated with an increased risk of ovarian carcinoma.

## 1. Introduction

The system of DNA repair takes part in maintaining the genomic integrity which undergoes changes under exo- and endogenous factors. There were more than 130 DNA repair genes identified, in which a series of single nucleotide polymorphisms (SNPs) were discovered [1]. In order to define the role, which may be played by these variants in modulating the risk of cancer formation, it is necessary to define their functional significance. The variability, perceived in DNA repair genes, may be of clinical importance for evaluation of the risk of occurrence of a given type of cancer and its prophylactics and therapy.

The repair process usually encompasses two stages: the excision of lesion and the repair synthesis. This is how the repair system acts via base-excision repair (BER), nucleotide excision repair (NER), and mismatch repair (MMR). Totally converse is the repair system activity by direct lesion reversal, in which there is merely a single-stage process with maintained integrity of the DNA phosphodiester chain and the system of recombination repair (HR).

A NER system removes short DNA oligonucleotides containing a damaged base [2]. NER recognizes bulky lesions caused by carcinogenic compounds and covalent linkages between adjacent pyrimidines resulting from UV exposure. NER is a multistep process involving multiple proteins such as ERCC1, ERCC2, ERCC3, ERCC4, PCNA, RPA, XPA, and p53. Because NER is involved in removing a substantial amount of DNA damage, which can contribute to the genome instability, it is reasonable to check whether variability in the genes coding for NER products may be associated with ovarian cancer.

Xeroderma pigmentosum complementation group D (*XPD*), also called excision repair cross-complimentary group 2 (*ERCC2*), is one of the most important low-penetrant genes which is located at chromosome 19q13.3 and involved in the nucleotide excision repair (NER) pathway and removes certain DNA cross-links, ultraviolet photolesions, and bulky chemical adducts.

*ERCC2* gene is highly polymorphic. An A → C polymorphism in* ERCC2* codon 751 of exon 23 leads to Lys → Gln amino acid substitution (Lys751Gln, rs13181) that is associated with a DNA damage repair phenotype.

Many epidemiological studies with the aim of identifying the role of* ERCC2* polymorphisms in the risk of various cancers have been done and different association between Lys751Gln polymorphism and the risk of lung cancer [3–5], glioma [6], colorectal cancer [7], breast cancer [8, 9], and esophageal squamous cell carcinoma [10] has been reported.

In the reported study, the interest of the authors was focused onto the studies of the Lys751Gln polymorphism of* ERCC2* gene in a group of patients with ovarian cancer and in a group of healthy people. The Lys751Gln polymorphism of* ERCC2* gene was selected on the basis of literature data, which are highly suggestive of its correlations with ovarian cancer [11–13]. In patients with ovarian cancer, evaluated by Wu et al. and Kang et al., the Lys751Gln polymorphism was associated with cancer development [11, 12]. In ovarian cancer subjects, described by Vella et al., polymorphisms in the* ERCC2 *gene (Lys751Gln and Asp312Asn) positively affect the response to therapy with carboplatin/paclitaxel [13]. Therefore, more research is needed to better understand the possible biological mechanisms of development and the role of Lys751Gln polymorphism in this rare, neoplastic transformation process.

The aim of this study was to analyze the frequency of alleles and genotypes of Lys751Gln (rs13181) in* ERCC2* with an attempt to determine the impact this polymorphism exerts on ovarian cancer.

## 2. Materials and Methods

### 2.1. Patients

Formalin-fixed paraffin-embedded (FFPE) tumour tissue specimens were obtained from women (*n* = 430) with ovarian carcinoma, treated at the Department of Surgical Gynaecology and Gynaecologic Oncology, Institute of Polish Mothers Memorial Hospital, between 2000 and 2012. We enrolled only women born and living in central Poland (Łódź region). The distribution of sociodemographic features of the study participants is shown in Table 1. Study subjects were interviewed using questionnaire that included sociodemographics, medical history, health related information, alcohol consumption, smoking status, menstrual and reproductive histories, and hormone replacement therapy (HRT) use. All of the studied individuals were Caucasians and constituted a homogenous population from the same ethnic and geographical origins. Body mass index (BMI) was calculated based on current weight in kilograms divided by height in meters squared. Drinking habits were categorized in terms of never/rare drinkers, ex-drinkers, or current drinkers who consumed 1–8.9 U/week (light drinkers), 9–17.9 U/week (moderate drinkers), or 18 U/week (heavy drinkers), where 1 U = 22 g ethanol. Participants were divided into four groups: nonsmokers (never), subjects smoking 10 cigarettes per day for 10 years (ever), those smoking 20 cigarettes per day for 20 years (moderate), and those smoking 20 cigarettes per day for 30 years (heavy). A positive family history of ovarian cancer was defined as reporting of ovarian cancer in one or more first-degree relatives. None of the recruited patients received preoperative chemo- or radiotherapy. The age of the patients ranged from 38 to 81 years (the mean age 53.2 ± 10.11). All the diagnosed tumours were graded by criteria of the International Federation of Gynaecology and Obstetrics (FIGO). DNA from normal ovarian tissue (*n* = 430) served as control (age range 39–83, mean age 50.31 ± 18.21). They were nonrelated women that have never been diagnosed with ovarian tumors, other tumors, or chronic disease and were randomly selected and frequency matched to the cases on age. The Local Ethical Committee approved the study and each patient gave a written consent for participation in the study.

### 2.2. DNA Isolation

Genomic DNA was prepared using QIAamp DNA FFPE Tissue Kit (Qiagen GmbH, Hilden, Germany) according to the manufacturer instruction.

### 2.3. Genotype Determination

The PCR-restriction fragment length polymorphism (PCR-RFLP) method was used to detect the genotypes of the Lys751Gln polymorphisms as described previously [14].

Primers (forward: 5′-CTGCTCAGCCTGGAGCAGC-3′, reverse: 5′- TAGAATCAGAGAGAGGAGACGCTG-3′) were applied to assess SNP Lys751Gln (rs13181). The 25 *μ*L PCR mixture contained about 100 ng of DNA, 12.5 pmol of each primer, 0.2 mmol/L of dNTPs, 2 mmol/L of MgCl_2_, and 1 U of Taq DNA polymerase (TaKaRa, Japan). PCR products were electrophoresed in a 2% agarose gel and visualised by ethidium bromide staining. All PCR was carried out in a DNA Thermal Cycler PTC-100 TM (MJ Research, Inc., Waltham, MA, USA). After an initial denaturation at 95°C for 5 min, 35 cycles of amplification with denaturation at 95°C for 30 s, annealing at 62°C for 30 s, and extension at 72°C for 30 s were performed, followed by a final extension step of 7 min at 72°C. The PCR product was digested overnight with 1 U of* Pst*I (Fermentas, Vilnius, Lithuania) at 37°C. The cleavage with* Pst*I produced fragments of 161, 161/120/41, and 120/41 bp corresponding to the Lys/Lys, Lys/Gln, and Gln/Gln genotypes of the* ERCC2* gene, respectively.

### 2.4. Statistical Analysis

The observed numbers of each* ERCC2* genotype were compared with those expected for a population in Hardy-Weinberg equilibrium by using the Chi-square (*χ* ^2^) test. Genotype and allele frequencies in cases and controls were compared by *χ* ^2^ test. Logistic regression analysis was used to compute odds ratio (OR) and associated 95% confidence interval (95% CI) relating Lys751Gln SNP as well as combinations of Lys751Gln SNP and other analysed factors presented in Table 1 to the risk of ovarian cancer. *p* values < 0.05 were considered significant. All the statistical analyses were performed, using the STATISTICA 6.0 software (StatSoft, Tulsa, Oklahoma, USA).

## 3. Results

The distribution of genotypes and the frequency of alleles of* ERCC2* gene in patients and controls are presented in Table 2. An association (OR 5.01; 95% CI 3.37–7.43; *p* < 0.0001) was found between the Gln/Gln genotype of the Lys751Gln polymorphism of* ERCC2* gene and ovarian cancer occurrence. Variant 751Gln allele of* ERCC2 *increased cancer risk (OR 3.61; 95% CI 2.92–4.45; *p* < 0.0001).

The observed genotype frequencies of* ERCC2* Lys751Gln SNP in the controls were in agreement with Hardy-Weinberg equilibrium (*p* > 0.05), but the observed genotype frequencies of* ERCC2* Lys751Gln SNP in patients were not in agreement with Hardy-Weinberg equilibrium (*p* < 0.05). It is caused by the very low abundance of the Lys/Lys genotype in the examined Polish population.

Histological grading was related to* ERCC2* polymorphism. Histological grades were evaluated in all the cases (*n* = 430). Grades 2 and 3 were accounted for together for statistical analysis (see Table 3). An increase was observed, regarding Gln/Gln homozygote frequency (OR 6.96; 95% CI 3.41–14.21; *p* < 0.0001) in grade 1 patients, according to FIGO criteria. Moreover, ovarian cancer patients in G1 had an overrepresentation of Gln allele (OR 4.98; 95% CI 3.37–7.40; *p* < 0.0001).

Clinical FIGO staging was also related to* ERCC2* Lys751Gln polymorphism (Table 4). An increase was observed, regarding Gln/Gln homozygotes frequency (OR 9.96; 95% CI 4.40–22.60; *p* < 0.0001) in stage I patients, according to FIGO classification. A tendency for an increased risk of ovarian cancer progression was observed with the occurrence of Gln allele (OR 6.59; 95% CI 4.27–10.19; *p* < 0.0001) of* ERCC2* polymorphism.

Our data did not demonstrate any statistically significant correlation between* ERCC2* polymorphisms and the risk factors for ovarian cancer, such as BMI (body mass index), smoking status, alcohol consumption, family history of cancer, pregnancy, ascites, HRT, size of tumour, menarche, and the women with ovarian cancer (“data not shown”).

## 4. Discussion

An attempt was undertaken in the presented study to determine whether single nucleotide polymorphism in the DNA repair pathway (*ERCC2*-Lys751Gln) was associated with the risk of ovarian cancer in Polish women. DNA is regularly damaged by endogenous and exogenous mutagens. The genes involved in DNA repair and in the maintenance of genome integrity play a crucial role in providing protection against mutations that may lead to cancer [15–19]. The DNA repair pathways play a vital role in protecting against gene mutation caused by carcinogenesis, among which the nucleotide excision repair (NER) pathway is one of the important DNA repair systems. The nucleotide excision repair system removes short DNA, damaged base-containing oligonucleotides [2]. NER is a multistep process, involving numerous proteins, and is classified into global genome repair (GG-NER), which occurs in the genome, and transcription-coupled repair (TCR), which removes lesions in the transcribed strand of active genes. The* ERCC1* gene is important in repairing DNA damage and genomic instability and is involved in the nucleotide excision repair pathway. Single nucleotide polymorphism Lys751Gln (rs13181) is one of the most widely studied genetic markers in* ERCC2* and its role in various cancers' development is evident [20]. Exchange of 751 Lys for Gln in the* ERCC2* gene can lead to a conformational change in the encoded protein at the domain of the interaction between ERCC2 and its helicase activator, p44, inside the TFIIH Complex [21]. The Gln/Gln genotype has been associated with an increased risk of lung carcinoma [5] and correlated with higher risk of breast, bladder, and skin cancer [8, 9, 22, 23]. Single nucleotide polymorphisms, as important genetic biomarkers, have been reported to be related to altered gene expression and protein activity.* ERCC2* Lys751Gln SNP is associated with suboptimal DNA repair capacity [24, 25]. From the current point of view, it seems to be more important to analyze the studies focusing on the effect of ERCC2 Lys751Gln polymorphisms in ovarian cancer. Some studies have suggested that low* ERCC2* expression is associated with increased chemotherapeutic sensitivity and thus considered a predictive marker for patients with ovarian cancer receiving combination gemcitabine and cisplatin chemotherapy [13]. The researchers found also the association between* ERCC2* Lys751Gln polymorphism and lower DNA repair capacity. The time to ovarian cancer progression was significantly higher in gemcitabine/cisplatin-treated patients with the Lys751Gln genotype than in those with the Lys751Lys genotype [13]. Vella et al. reported that the combination of* ERCC1* and* ERCC2* genotype is associated with risk of ovarian carcinoma [13]. The Chinese researchers found also the association between ovarian cancer and* ERCC2* Lys751Gln polymorphism [11]. Yet, to our knowledge, there are no reports that assess the effect of this genetic alteration on the risk of ovarian cancer in Polish population.

Our results indicate that the Lys751Gln polymorphism of* ERCC2* gene deserves particular attention. In the studies on a series of 430 DNA samples from patients with ovarian cancer, originating from an ethnically homogenous population, we found a relationship of the studied polymorphisms with endometrial cancer occurrence. We demonstrated that 751Gln allele was associated with risk for the neoplasm. The* ERCC2*-Gln/Gln genotype increased 5 times the risk of ovarian cancer formation. Additionally, some important connections with grading (G1) and staging (SI) of ovarian carcinoma were presented. Our study was performed on an ethnically homogenous population, which may improve our knowledge, regarding to what extent the genotype-phenotype relationship variations are population related.

Our results are in line with the data from other reports, introducing an important role of* ERCC2* Lys751Gln polymorphism in ovarian carcinoma occurrence. Similar to our observation, the recent reports demonstrate that* ERCC2* Lys751Gln genotype seems to be associated with an elevated ovarian cancer risk [11].

In conclusion, our results indicate that the* ERCC2* Lys751Gln SNP may be involved in the susceptibility of ovarian cancer in the Polish population. Further research on SNP in ovarian carcinoma is warranted to obtain more conclusive outcomes.

## Figures and Tables

**Table 1:** Characteristics of the study population (*n* = 430).

Characteristics	Number of cases (%)
*Histology of tumour*	
Serous	148 (34.4)
Mucinous	20 (4.7)
Endometrioid	120 (27.9)
Clear cell	23 (5.3)
Undifferentiated	103 (24.0)
Other	16 (3.7)

*Number of pregnancies*	
1	196 (46)
2-3	152 (35)
>4	82 (19)

*Body mass index*	
<19 0 0	34 (8.0)
18–25 25 37	260 (60.4)
26–29 40 50	98 (22.8)
>30	38 (8.8)

*Smoking status*	
Never	99 (23)
Ever	172 (40)
Moderate	60 (14)
Heavy	99 (23)

*Alcohol intake*	
Never/rare	132 (31)
Light	108 (25)
Moderate	60 (14)
Heavy	64 (15)
Ex-drinker	66 (15)

*Family history of ovarian cancer*	
Yes	150 (35)
No	280 (65)

*Ascites*	
Present	153 (36)
Absent	277 (64)

*Use of hormone replacement therapy (HRT)*	
Yes	293 (8)
No	137 (32)

*Size of tumor*	
>5 cm	270 (63)
<5 cm	160 (37)

*Tumour wall infiltration/injury*	
Present	129 (30)
Absent	301 (70)

*Menarche*	
<12 years old	290 (67)
>12 years old	140 (33)

*Grading*	
G1	200 (47)
G2	220 (51)
G3	10 (2)

*Staging*	
I	190 (44)
II	230 (54)
III	10 (2)

**Table 2:** Genotypes and alleles distributions of SNP Lys751Gln in *ERCC2* in ovarian cancer cases versus lean controls.

*ERCC2*-Lys751Gln	Patients (*n* = 430)		Controls (*n* = 430)		OR (95% CI)^a^	*p* ^b^
	Number	(%)	Number	(%)		
Lys/Lys	62	14.4	96	22.3	1.00 Ref.	
Lys/Gln	64	14.9	240	55.8	0.41 (0.27–0.62)	<0.0001
Gln/Gln	304	70.7	94	21.8	5.01 (3.37–7.43)	<0.0001
Lys	188	21.8	432	50.2	1.00 Ref.	
Gln	672	78.2	428	49.8	3.61 (2.92–4.45)	<0.0001

^a^Crude odds ratio (OR); 95% CI: confidence interval at 95%; ^b^Chi square.

**Table 3:** Dependence of *ERCC2 *gene polymorphism genotypes and allele frequency on tumour grade in patients with ovarian cancer^a^.

Grade^b^	Ovarian cancer patients			
	G1 (*n* = 200)	G2 + G3 (*n* = 230)	OR (95% CI)^c^	*p* ^d^
*ERCC2*-Lys751Gln	Number (%)	Number (%)		
Lys/Lys	10 (5%)	52 (22.6%)	1.00 Ref.	
Lys/Gln	16 (8%)	48 (20.9%)	1.73 (0.71–4.18)	0.312
Gln/Gln	174 (87%)	130 (56.5%)	6.96 (3.41–14.21)	<0.0001
Lys	36 (9%)	152 (33%)	1.00 Ref.	
Gln	364 (91%)	308 (67%)	4.98 (3.37–7.40)	<0.0001

^a^ *n* = 430; ^b^according to FIGO criteria; ^c^crude odds ratio (OR); 95% CI: confidence interval at 95%; ^d^Chi square.

**Table 4:** Dependence of genotypes and frequencies of *ERCC2 *gene polymorphism alleles on tumor stage in ovarian cancer patients^a^.

Stage	SI (*n* = 190)	SII + SIII (*n* = 240)	OR (95% CI)^b^	*p* ^c^
*ERCC2*-Lys751Gln	Number (%)	Number (%)		OR (95% CI)
Lys/Lys	7 (3.7%)	55 (23%)	1.00 Ref.	
Lys/Gln	13 (6.8%)	51 (21.2%)	2.00 (0.74–5.42)	0.254
Gln/Gln	170 (89.5%)	134 (55.8%)	9.96 (4.40–22.60)	<0.0001
Lys	27 (7.1%)	161 (33.5%)	1.00 Ref.	
Gln	353 (92.9%)	319 (66.5%)	6.59 (4.27–10.19)	<0.0001

^a^ *n* = 430; ^b^crude odds ratio (OR); 95% CI: confidence interval at 95%; ^c^Chi square.

## References

[1] Wood R. D., Mitchell M., Sgouros J., Lindahl T. (2001). Human DNA repair genes. *Science*.

[2] Hanawalt P. C. (2002). Subpathways of nucleotide excision repair and their regulation. *Oncogene*.

[3] Tan X., Xian L., Chen X. (2014). Association between ERCC2 Lys751Gln polymorphism and lung cancer risk: a meta-analysis involving 23,370 subjects. *Twin Research and Human Genetics*.

[4] Yin J., Vogel U., Ma Y., Guo L., Wang H., Qi R. (2006). Polymorphism of the DNA repair gene *ERCC2* Lys751Gln and risk of lung cancer in a northeastern Chinese population. *Cancer Genetics and Cytogenetics*.

[5] De Ruyck K., Szaumkessel M., De Rudder I. (2007). Polymorphisms in base-excision repair and nucleotide-excision repair genes in relation to lung cancer risk. *Mutation Research—Genetic Toxicology and Environmental Mutagenesis*.

[6] Hui L., Yue S., Gao G., Chang H., Li X. (2014). Association of single-nucleotide polymorphisms in ERCC1 and ERCC2 with glioma risk. *Tumour Biology*.

[7] Ni M., Zhang W.-Z., Qiu J.-R. (2014). Association of ERCC1 and ERCC2 polymorphisms with colorectal cancer risk in a Chinese population. *Scientific Reports*.

[8] Smolarz B., Makowska M., Samulak D. (2014). Single nucleotide polymorphisms (SNPs) of *ERCC2*, *hOGG1*, and *XRCC1* DNA repair genes and the risk of triple-negative breast cancer in Polish women. *Tumor Biology*.

[9] Brewster A. M., Jorgensen T. J., Ruczinski I. (2006). Polymorphisms of the DNA repair genes XPD (Lys751Gln) and XRCC1 (Arg399Gln and Arg194Trp): relationship to breast cancer risk and familial predisposition to breast cancer. *Breast Cancer Research and Treatment*.

[10] Zhang Y., Wang L., Wang P. (2014). Association of single nucleotide polymorphisms in ERCC2 gene and their haplotypes with esophageal squamous cell carcinoma. *Tumor Biology*.

[11] Wu K.-G., He X.-F., Li Y.-H., Xie W.-B., Huang X. (2014). Association between the XPD/ERCC2 Lys751Gln polymorphism and risk of cancer: evidence from 224 case-control studies. *Tumor Biology*.

[12] Kang S., Sun H.-Y., Zhou R.-M., Wang N., Hu P., Li Y. (2013). DNA repair gene associated with clinical outcome of epithelial ovarian cancer treated with platinum-based chemotherapy. *Asian Pacific Journal of Cancer Prevention*.

[13] Vella N., Aiello M., Russo A. E. (2011). ‘Genetic profiling’ and ovarian cancer therapy. *Molecular Medicine Reports*.

[14] Sobczuk A., Poplawski T., Blasiak J. (2012). Polymorphisms of DNA repair genes in endometrial cancer. *Pathology and Oncology Research*.

[15] Jackson S. P. (2002). Sensing and repairing DNA double-strand breaks. *Carcinogenesis*.

[16] Helleday T. (2003). Pathways for mitotic homologous recombination in mammalian cells. *Mutation Research: Fundamental and Molecular Mechanisms of Mutagenesis*.

[17] Khanna K. K., Jackson S. P. (2001). DNA double-strand breaks: signaling, repair and the cancer connection. *Nature Genetics*.

[18] Wilson D. M., Bohr V. A. (2007). The mechanics of base excision repair, and its relationship to aging and disease. *DNA Repair*.

[19] Jiricny J., Nyström-Lahti M. (2000). Mismatch repair defects in cancer. *Current Opinion in Genetics and Development*.

[20] Wu K.-G., He X.-F., Li Y.-H., Xie W.-B., Huang X. (2014). Association between the XPD/ERCC2 Lys751Gln polymorphism and risk of cancer: evidence from 224 case–control studies. *Tumor Biology*.

[21] Fan L., Fuss J. O., Cheng Q. J. (2008). XPD helicase structures and activities: insights into the cancer and aging phenotypes from XPD mutations. *Cell*.

[22] Stern M. C., Conway K., Li Y., Mistry K., Taylor J. A. (2006). DNA repair gene polymorphisms and probability of p53 mutation in bladder cancer. *Molecular Carcinogenesis*.

[23] Appelbaum K. M., Karagas M. R., Hunter D. J. (2007). Polymorphisms in nucleotide excision repair genes, arsenic exposure, and non-melanoma skin cancer in New Hampshire. *Environmental Health Perspectives*.

[24] Lunn R. M., Helzlsouer K. J., Parshad R. (2000). XPD polymorphisms: effects on DNA repair proficiency. *Carcinogenesis*.

[25] Duell E. J., Wiencke J. K., Cheng T.-J. (2000). Polymorphisms in the DNA repair genes *XRCC1* and *ERCC2* and biomarkers of DNA damage in human blood mononuclear cells. *Carcinogenesis*.

